# Evidence for Reconsidering the Taxonomic Status of Closely Related *Oligonychus* Species in *punicae* Complex (Acari: Prostigmata: Tetranychidae)

**DOI:** 10.3390/insects14010003

**Published:** 2022-12-21

**Authors:** Hafiz Muhammad Saqib Mushtaq, Muhammad Kamran, Amgad A. Saleh, Fahad Jaber Alatawi

**Affiliations:** 1 Acarology Research Laboratory, Department of Plant Protection, College of Food and Agriculture Sciences, King Saud University, P.O. Box No. 2460, Riyadh 11451, Saudi Arabia; 2 Plant Pathology Laboratory, Department of Plant Protection, College of Food and Agriculture Sciences, King Saud University, P.O. Box No. 2460, Riyadh 11451, Saudi Arabia

**Keywords:** aedeagus, cryptic species, DNA barcoding, integrative taxonomy, spider mites

## Abstract

**Simple Summary:**

The present study comprehensively addressed a taxonomic problem in the Oligonychus punicae species complex. Based on the morphological and molecular data, two spider mite species, namely Oligonychus mangiferus (Rahman and Sapra) and O. vitis (Zaher and Shehata) are synonymized with O. punicae (Hirst). Moreover, DNA-based analysis showed that there are GenBank COI and ITS2 sequences of Oligonychus that need to be revisited to confirm the identity of their voucher specimens. The importance of an integrative taxonomic approach is discussed for the accurate identification of closely related Oligonychus species.

**Abstract:**

To elucidate the taxonomic problems in species delineation within the *Oligonychus punicae* complex (*O. punicae, O. mangiferus*, and *O. vitis*) (Acari: Prostigmata: Tetranychidae), we performed morphological and molecular investigations on mite samples, collected from different hosts/countries. Thirty-nine samples of *punicae* complex, collected from Egypt, Pakistan, and Saudi Arabia (SA), did not show any considerable morphological differences in females and males. All 39 samples of the *punicae* complex resembled the original description of *O. punicae*, while the claimed Mexican *O. punicae* was distinctively different based on male aedeagus. Molecularly, the low nucleotide diversity ranged from 0% to 2.1% (ITS2-rDNA) and 0% to 1% (COI-mtDNA), and was observed among various DNA sequences of the *punicae* complex from Egypt, India, Israel, Pakistan, and SA, confirming their identity as one species. The high genetic divergence ranged from 17.2% to 18.8% (ITS2) and 9.2% to 10.2% (COI), observed between the claimed Mexican *O. punicae* and all other sequences of the *punicae* complex, indicating that the Mexican sample do not belong to *O. punicae*. Basing our findings on both morphological and molecular data, we can conclude that *O. mangiferus* and *O. vitis* are synonymized with *O. punicae*. Additionally, this study reveals that the claimed Mexican *O. punicae* needs to be re-identified.

## 1. Introduction

*Oligonychus* Berlese is the largest genus of spider mites in the family Tetranychidae Donnadieu (Acari: Prostigmata), with 212 species that have been reported so far [1,2]. Differentiation of *Oligonychus* species is difficult and usually depends on the morphology of the male aedeagus [3,4]. However, both adult sexes are compulsory for the identification of the *Oligonychus* species. Indeed, exact species identification is usually challenging because of the minute differences in male aedeagus and the limited potential diagnostic characteristics available in females of several *Oligonychus* species [3,4,5,6,7]. Moreover, the key differential character of the male aedeagus becomes unreliable with improperly mounted specimens (not in a lateral position) or brief morphological descriptions without illustrations of the aedeagus [8,9]. Recently, a comprehensive taxonomic assessment of *Oligonychus* revealed the presence of various doubtful species [1].

In the genus *Oligonychus,* the *coffeae* species subgroup (=*ununguis* and *bicolor* subgroups, sensu Pritchard and Baker) was characterized by Mushtaq et al. and assigned 44 species [1]. This subgroup comprises many related *Oligonychus* species with similar morphological characteristics [4,10]. Therefore, separation among species of this subgroup is usually based on slight differences in aedeagus shape, the size of the female palp spinneret, and is occasionally limited to the inhabited host plant [3,4,10]. Among the members of the *coffeae* subgroup, three pestiferous *Oligonychus* species, the avocado brown mite *O. punicae* (Hirst), the mango red spider mite *O. mangiferus* (Rahman and Sapra), and the table grape red mite *O. vitis* (Zaher and Shehata) are mainly distinguished based on minute differences in aedeagal morphology [3,4,7,11,12,13,14,15,16,17]. However, Khanjani et al. considered these aedeagal differences to be “subtle variations” and highlighted the fact that these species are part of the “greatest taxonomic problem” [10] in the *coffeae* subgroup.

All three closely related *Oligonychus* species of *O. punicae, O. mangiferus*, and *O. vitis* were reported on the common type of host plant, *Vitis vinifera* L. (Vitaceae) [9,18,19]. Compared to *O. vitis,* the species *O. punicae* and *O. mangiferus* have been recorded on numerous similar hosts in different common localities [20]. However, both of these *Oligonychus* species were originally reported in neighbouring type localities: *O. punicae* was reported in Coimbatore, India [18], and *O. mangiferus* was reported in Faisalabad, Pakistan [9].

Morphotaxonomic studies have highlighted the existence of sibling and cryptic species within *Oligonychus*, and referred to species complexes, e.g., *coffeae* complex, *punicae* complex, *pratensis* complex, *pritchardi* complex, *perseae* complex, *subnudus* complex, *sacchari* complex, and *ununguis* complex [1,3,4,5,6,7,10,21,22,23,24,25]. Such species complexes are difficult to resolve using morphology alone [4]. Therefore, an integrated taxonomic approach using morphological and molecular/biological data is essential to differentiate among/between the closely related *Oligonychus* species [10,26,27,28].

DNA-based markers, e.g. the internal transcribed spacer region ITS2 of the nuclear ribosomal DNA and the mitochondrial cytochrome *c* oxidase subunit I (COI) gene have been applied accurately together with morphological data for the identification and confirmation of morphologically related tetranychid species [26,28,29,30,31,32]. The application of integrative taxonomy for species delineation has resolved many problems of synonymy and the misidentification of closely related species in the family Tetranychidae [33,34,35]. Therefore, the main objective of the present study was to apply an integrative taxonomical approach to investigating the species status of the three closely related *Oligonychus* species belonging to the *punicae* species complex, *O. punicae*, *O. mangiferus*, and *O. vitis*.

## 2. Materials and Methods

### 2.1. Spider Mite Samples Collection, Preservation, and Processing

**Figure 1:**
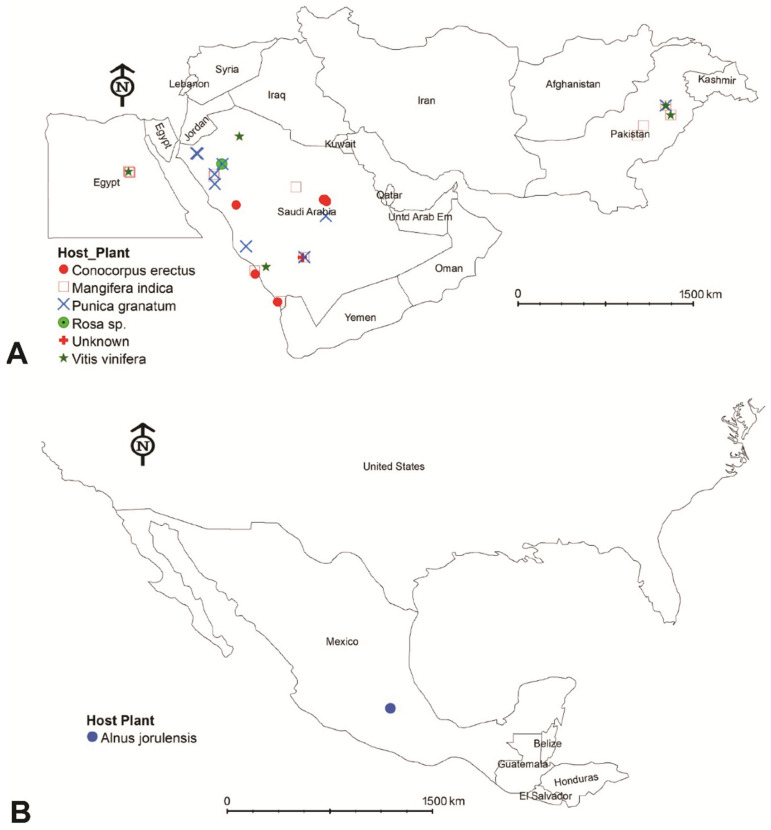
Geographical distribution of the (**A**) 39 spider mite samples of the *punicae* complex along with the (**B**) sample of claimed Mexican *Oligonychus punicae* [37], collected from Egypt, Mexico, Pakistan and Saudi Arabia and seven host plants (*Alnus jorulensis*, *Conocorpus erectus*, *Mangifera indica*, *Punica granatum*, *Rosa* sp., Unknown and *Vitis vinifera*) in the present study (ARCGIS 10.5., esri.com computer software, Esri^TM^, Redlands, CA, USA).

**Figure 2:**
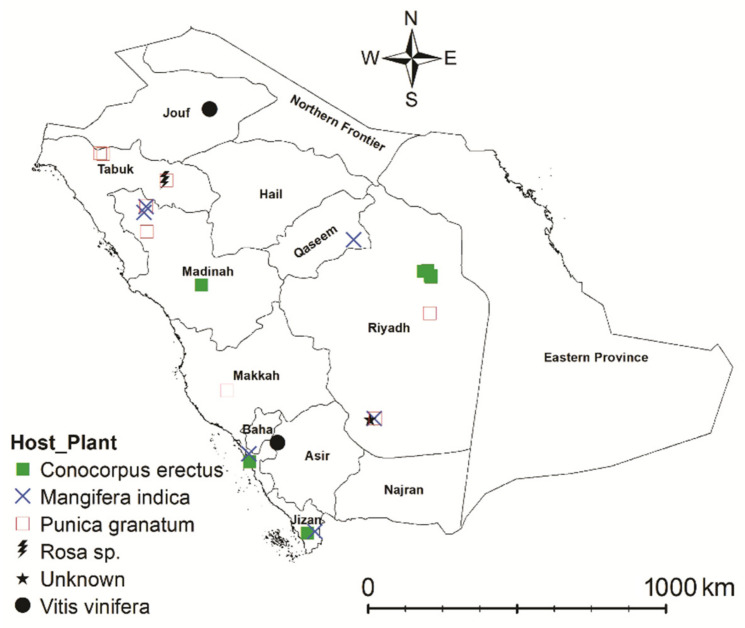
Geographical distribution of *Oligonychus punicae* in Saudi Arabia, reported on six host plants (*Conocorpus erectus*, *Mangifera indica*, *Punica granatum*, *Rosa* sp., Unknown & *Vitis vinifera*) and collected from 26 localities of eight provinces (Asir, Jouf, Jizan, Makkah, Madinah, Qassim, Riyadh, & Tabuk) (ARCGIS 10.5., esri.com computer software, Esri^TM^, Redlands, CA, USA).

Forty spider mite samples from the *punicae* complex were collected from seven host plants in different seasons from various localities within four countries (Figure 1; Table S1). Most of the samples were collected during the period from 2017 to 2022, except for a sample (voucher no.: 105; Table S1) that had been previously collected in 2012 and was identified as *O. punicae* [36] from Saudi Arabia (SA). A total of 26 samples (voucher no.: 1, 5, 6, 13, 14, 21, 26, 27, 37, 43, 44, 56, 59, 62, 63, 64, 70, 76, 77, 78, 80,104, 105, 134, 137 and 147; Table S1) were collected from various localities within eight SA provinces of Asir, Jouf, Jizan, Makkah, Madinah, Qassim, Riyadh, and Tabuk (Figure 2). Moreover, eight samples (voucher no.: 48, 71, 72, 73, 74, 75, 184, and 185; Table S1) were collected from four different localities and three hosts in Pakistan. Additionally, five samples of *O. mangiferus* (voucher no.: 42, 178, 180, 181, and 183) and an *Oligonychus* sp. (voucher no.: 52, previously claimed as *O. punicae*) [37] were brought from Egypt and Mexico, respectively (Table S1). Some of the collected samples (voucher no.: 6, 27, 70, 72, 74, 75, 78, 104, 134, 137, 147, 184, and 185; Tables S1 and S2) only contained female specimens. The collection details (i.e., collection date, locality, host plant, GPS coordinates, and collector name) of each sample are provided in Table S1. All collected specimens were deposited at the King Saud University Museum of Arthropods (Acarology section), Department of Plant Protection, College of Food and Agriculture Sciences, King Saud University (KSU), Riyadh, Saudi Arabia.

Both adult females and males were mounted on glass slides in Hoyer’s medium under a SZX10 stereomicroscope (Olympus, Tokyo, Japan). Samples were dried in an oven at 30–40 °C for five days. Male aedeagi from the *punicae* complex samples were imaged using an auto-montage software system (Syncroscopy, Cambridge, UK) attached to a DM2500 phase contrast microscope (Leica, Wetzlar, Germany) and then drawn using Adobe Illustrator software (Adobe Systems Inc., San Jose, CA, USA).

### 2.2. Morphological Study

Morphological investigations were performed using a BX51 fluorescence microscope (Olympus, Tokyo, Japan) in the Acarology Laboratory, KSU, for all 40 spider mite samples either collected from Egypt, Mexico, Pakistan, or SA (Table S1). In fact, the samples from Egypt (*O. mangiferus*), Mexico (*O. punicae*), and Pakistan (*O. mangiferus*) were initially identified/labelled and sent to us by local Acarologists [37,38] and our colleagues at our request.

**Figure 3:**
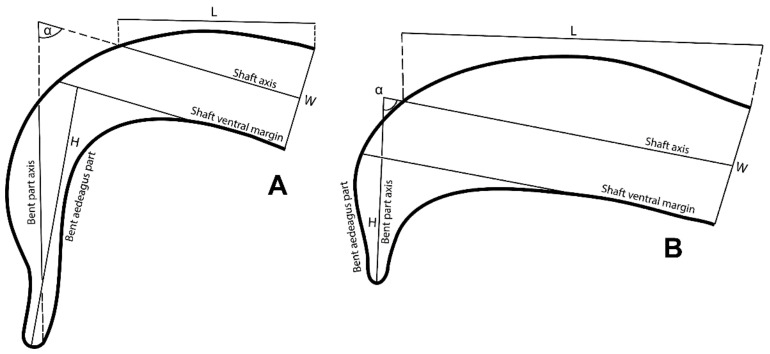
Morphological parameters (H: the height of the bent aedegal part; L: the length of the shaft dorsal margin; W: the width of the shaft, and α: the angle formed between the shaft axis and the axis of the bent part) that was measured for comparison of the key differential character of the male aedeagus, among different collected spider mite samples, representing the (**A**) claimed Mexican *Oligonychus punicae* (bent part tip bends posteriorly), and (**B**) *O*. *punicae* (bent part tip straight).

Slides with mounted female and male (both dorsal and lateral position) specimens were comprehensively investigated. Diagnostic keys were used to identify the genus [39], subgenus, species group, and subgroup [1]. For the species-level identification, various regionally prepared diagnostic keys were consulted [3,4,11,12,13,16,17,40,41,42,43], as well as a diagnostic key that was prepared recently for the identification of world *Oligonychus* species [44]. However, due to observed inconsistency and ambiguity in aedeagal characters that were used in previously published keys/descriptions, differentiation among members of the *punicae* complex (*O. punicae, O. mangiferus*, and *O. vitis*) was found to be unreliable. Therefore, to investigate the actual taxonomic status of the *punicae* complex, a formula was devised by measuring the aedeagal parameters/characters, e.g., length of the shaft dorsal margin (L, measured from the base of the shaft dorsal margin to the level at which the shaft axis line crosses the shaft dorsal margin, the point at which the shaft dorsal margin starts bending downward), the width of the shaft (W, maximum width of the shaft measured near the base of the shaft dorsal margin), the height of the bent aedeagal part (H, measured from the level of the shaft ventral margin to the tip of the bent part), and angle (α) formed between the shaft axis and axis of the bent part (Figure 3).

Moreover, we comprehensively reviewed the taxonomic literature and compared all collected samples with the original descriptions of *O. punicae* [18], *O. mangiferus* [9] and *O. vitis* [19], and their subsequent re-descriptions that were made from different geographical localities [3,4,5,7,10,11,12,13,14,17,25,41,45]. In addition, each sample was compared with the re-description (diagnosis) of *O. punicae* that was based on the type specimens (syntypes), which were observed by Dr. Jennifer J. Beard [46]. In addition, we tried to obtain and observe the type specimens of *O. mangiferus* and *O. vitis* with the help of acarologists in Pakistan and Egypt, respectively.

The types of *O. mangiferus* and *O. vitis* could not be observed in the present study, because either the information about the deposition of type specimens was missing in the original description of *O. mangiferus* [9], or types were lost as we were informed for *O. vitis.* However, eight samples (voucher no. 48, 71, 72, 73, 74, 75, 184, and 185; Table S1) from Pakistan including the specimens from the exact locality whence the original type of *O. mangiferus* was previously collected and described for the first time [9], and five samples from Egypt that were sent as *O. mangiferus* (voucher no. 42, 178, 180, 181, and 183; Table S1) were successfully analyzed. We also requested samples from Indian and Iranian acarologists, but did not receive the specimens of *O. punicae* from India (its type locality) and Iran.

### 2.3. Molecular Study

#### 2.3.1. DNA Extraction and Amplification of ITS2 and COI Regions

DNA was extracted from single adult females from 34 mite samples (Table S1) of the *punicae* complex using a DNeasy mini kit (Qiagen, Hilden, Germany), following the manufacturer’s guidelines. Six samples (voucher no. 75, 105, 178, 180, 181, and 183; Table S1) were not included in the molecular analysis due to the unavailability of sufficient specimens (Table S1). The concentration of the total genomic DNA solutions was assessed by a NanoDrop^TM^ One spectrophotometer (Thermofisher Scientific, Waltham, MA, USA). DNA samples were stored at −20 °C after the sample was labelled with the appropriate field information.

The ITS2-rDNA region was amplified from the 34 mite samples using PCR-primers, ITS2-forward (5′-GTCACATCTGTCTGAGAGTTGAGA-3′) and ITS2-reverse (5′-GTARCCTCACCTRMTCTGAGATC-3′) [29]. In addition, COI-forward primer (5′-TGATTTTTTGGTCACCCAGAAG-3′) and COI-reverse primer (5′-TACAGCTC CTATAGATA AAAC-3′) [32] were also used to amplify the COI-mtDNA fragments from 17 of the mite samples (Table S1). The PCR reaction was performed in a 30 μL reaction volume containing 15 μL 2× master mix (Molequle-On, Auckland, New Zealand), 0.4 μL of each 10 µM primer, 2 μL template DNA, and 12.2 μL Nuclease free water from Promega. The PCR conditions were as follows: a denaturation step at 94 °C for 5 min, followed by 35 cycles of a denaturation step at 94 °C for 60 s, an annealing step at 52 °C (for ITS2), and 53 °C (for COI) for 90 s and an extension step at 72 °C for 60 s, and a final extension step at 72 °C for 10 min. The PCR products were assessed in 1.2% agarose gel stained with acridine orange dye in 1× TAE buffer. Gels were observed and picturized using a *gel documentation* system (Uvitec, Cambridge, UK). For DNA sequencing, the PCR products were then purified using a Molequle-On PCR product purification kit (Molequle-On, Auckland, New Zealand).

#### 2.3.2. DNA Sequencing and Analysis

The purified products of the ITS2 and COI regions were directly sequenced using the same primers at the Macrogen sequencing facility (Macrogen Inc., Seoul, Republic of Korea). A total of 34 ITS2 and 17 COI sequences were obtained from spider mite samples of the *punicae* complex, representing different locations across four countries (Table S1). The sequences were cleaned and edited using Bioedit software [47]. The cleaned sequences were searched against the NCBI GenBank database using BLASTn. Based on the BLASTn results, homologous and closely related ITS2/COI sequences were retrieved from GenBank and aligned with their counterpart sequences obtained during the present study, using the CLUSTALW multiple alignment tool in Bioedit. The retrieved ITS2/COI sequences from GenBank represented *O. punicae, O. mangiferus*, and *O. vitis*, as well as some other closely related *Oligonychus* species including *O. amiensis* Ehara and Gotoh, *O. clavatus* Ehara, *O. castaneae* Ehara and Gotoh, *O. coffeae* (Nietner)*, O. camelliae* Ehara and Gotoh, *O. gotohi* Ehara, *O. hondoensis* Ehara, *O. ilicis* (McGregor), *O. karamatus* Ehara, *O. neocastaneae* Arabuli and Gotoh, *O. pustulosus* Ehara, *O. perditus* Pritchard and Baker, *O. tsudomei* Ehara, and *O. ununguis* (Jacobi). All ITS2/COI sequences of the *punicae* complex samples obtained during the present study were deposited in the NCBI-GenBank database (Table S1).

#### 2.3.3. Phylogenetic and Genetic Distances Analysis

Phylogenetic analyses were conducted to assess the genetic variations within and among different samples of *O. punicae, O. mangiferus, O. vitis* (the *punicae* complex), and their closely related *Oligonychus* species using MEGA-X [48]. Phylogenetic trees were constructed using the neighbour-joining (NJ) and the maximum likelihood (ML) methods of the Tamura–Nei model [49]. The robustness of the tree branches was tested with 1000 replications in a bootstrap analysis [50]. The ambiguous positions in the nucleotides were removed for each sequence pair using the pairwise deletion method. Furthermore, the pairwise *p*-distances (intraspecific and interspecific genetic divergence) were also calculated using MEGA-X [47].

## 3. Results

### 3.1. Morphological Analysis

**Figure 4:**
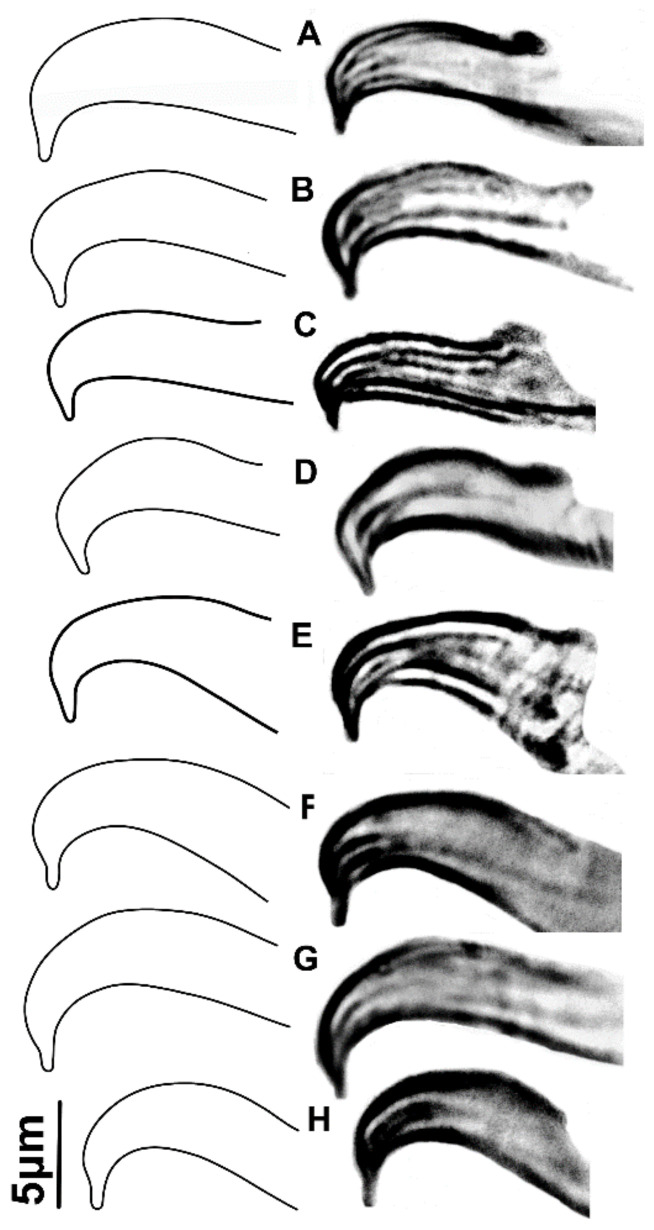
Illustrations/images of male aedeagi of different spider mite samples of the *punicae* complex; collected from Egypt {(**D**), voucher no. 42, *Mangifera indica*}, Pakistan {(**E**), voucher no. 71, *M. indica*—collected from the exact locality whence the original type of *Oligonychus mangiferus* was previously collected and described for the first time [9]; (**F**), voucher no. 73, *Punica granatum*}, and SA {(**A**), voucher no. 1, *M. indica*; (**B**), voucher no. 5 and (**H**), voucher no. 105, both *Conocorpus erectus*; (**C**), voucher no. 21, *P*. *granatum;* and (**G**), voucher no. 80, *Vitis vinifera*}.

The morphological investigations did not reveal any distinct differences in males and/or females from the 39 mite samples within the *punicae* complex (Tables S1 and S2)*,* regardless of the collection localities across three countries (Egypt, Pakistan and SA) and six host plants (*C. erectus*, *P. granatum*, *M. indica*, *Rosa* sp., unknown host and *V. vinifera*). All aedeagal morphological parameters (e.g., L, H, W and α; Figure 3) were quite similar, regardless of whether they were collected from Egypt (Table S2; Figure 4D), Pakistan (Table S2; Figure 4E,F), or SA (Table S2; Figure 4A–C,G–H). Moreover, the male aedeagus of the specimens of *O. mangiferus* (voucher no. 71, Tables S1 and S2; Figure 4E) collected from the exact locality whence the original type was previously collected and described for the first time [9] almost resembled the original description/illustration of *O. punicae* [18], and did not show any differences.

**Figure 5:**
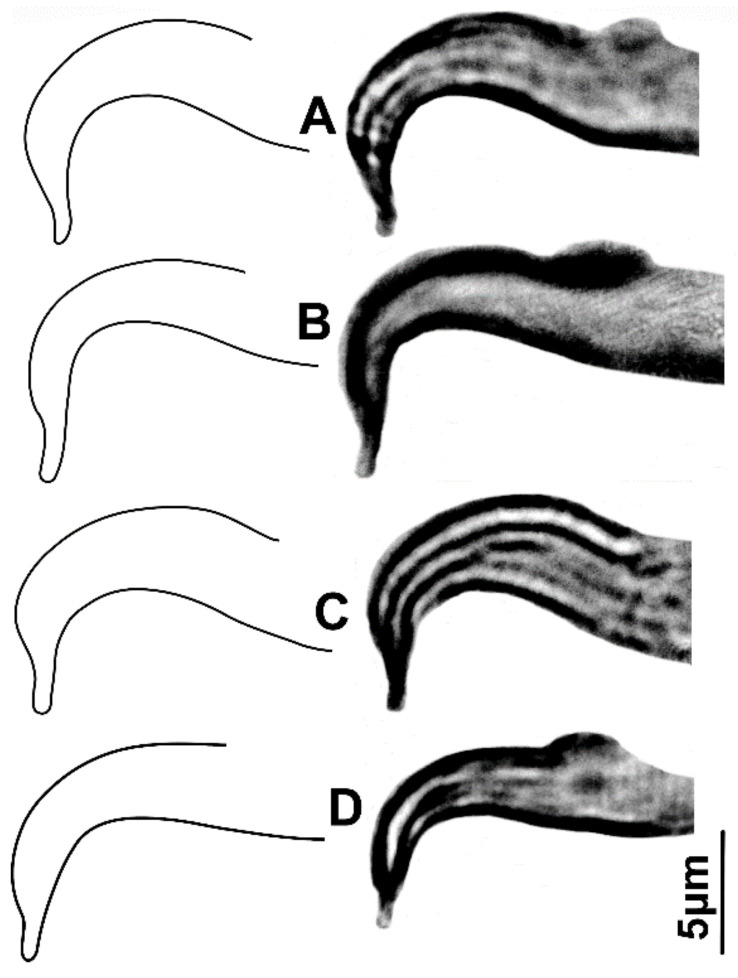
Illustrations/images of male aedeagi (**A**–**D**) drawn from four specimens of the claimed *Oligonychus punicae* population [37] collected from *Alnus jorulensis* in Mexico (voucher no. 52; Table S1), which needs to be re-identified.

Exceptionally, only the male aedeagus of the claimed Mexican *O. punicae* (voucher no. 52, Tables S1 and S2; Figure 5A–D) was consistently different from the other 39 samples (Tables S1 and S2; Figure 4); e.g., the bent aedeagal part (H), was sub-equal, equal or longer than the length (L) of the shaft dorsal margin (vs. one-quarter to less than three-quarters the length of the shaft dorsal margin; Table S2; Figure 4), and the bent aedeagal part was approximately 1.8 to 2.5 times longer (H) than the shaft width (W) (vs. 0.7 to 1.6 times longer than the shaft width, in all other 39 samples; Table S2; Figure 4).

### 3.2. Molecular Analysis

The estimated pairwise nucleotide *p*-distances for the ITS2 sequences within the *punicae* complex ranged from 0.000 and 0.021 (0% to 2.1%) (Table S3). However, the nucleotide *p*-distances of the ITS2 between the *punicae* complex and their counterparts in the claimed Mexican *O. punicae* ranged between 0.172 and 0.188 (17.2% to 18.8%), indicating that the Mexican specimens are not closely related to the *punicae* complex (Table S3).

**Figure 6:**
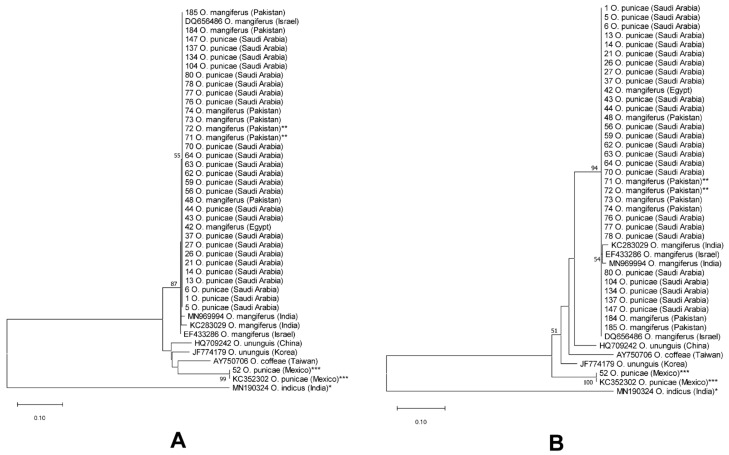
NJ (**A**) and ML (**B**) phylogenetic trees based on ITS2 sequences of 43 spider mite samples, representing different populations of four closely related *Oligonychus* species. A total of 25 ITS2 sequences were obtained/analyzed from different hosts and regions in Saudi Arabia, one from Egypt, and seven from Pakistan (** including two samples of *Oligonychus mangiferus* collected from the exact locality whence the original type was previously collected and described for the first time) [9]. Whereas 10 closely related ITS2 sequences were analyzed of *O. coffeae, O. mangiferus, O. ununguis*, and a cryptic *Oligonychus* species (*** previously claimed as *O. punicae* in Mexico) [37] that needs to be re-identified; in addition, *O. indicus* (* an *Oligonychus* species from the other subgenus *Reckiella* Tuttle and baker, used as an out-group taxon). Numbers on tree branches are bootstrap values obtained from 1000 replicates.

According to the ITS2-based NJ and ML phylogenetic trees (Figure 6), 37 sequences (samples) from the *punicae* complex from five countries (Egypt, India, Israel, Pakistan and SA) clustered together as a separate monophyletic clade with 87% and 94% bootstrap values, respectively (Figure 6). The sequences of the *punicae* complex were further separated into four haplotypes, namely H1 (Egypt, Israel, Pakistan and SA), H2 (Israel), H3 (India), and H4 (India) (Figure 6; Table S3). Interestingly, the two sequences of claimed Mexican *O. punicae* were distantly located at the tree bases (Figure 6).

The genetic distances using the COI also showed low divergence (from 0.000 to 0.010; 0% to 1%) among members of the *punicae* complex from Egypt, India, Pakistan, and SA (Table S4). In addition, there was a low genetic divergence between *O. vitis* (Accession no. MW517748; India) and the other two members (*O. punicae, O. mangiferus*) within the *punicae* complex (Table S4). However, the nucleotide *p*-distances of the COI between the *punicae* complex and their counterparts in the claimed Mexican *O. punicae* ranged between 0.092 and 0.102 (9.2% to 10.2%), indicating that the Mexican specimens are not closely related to the *punicae* complex (Table S4).

**Figure 7:**
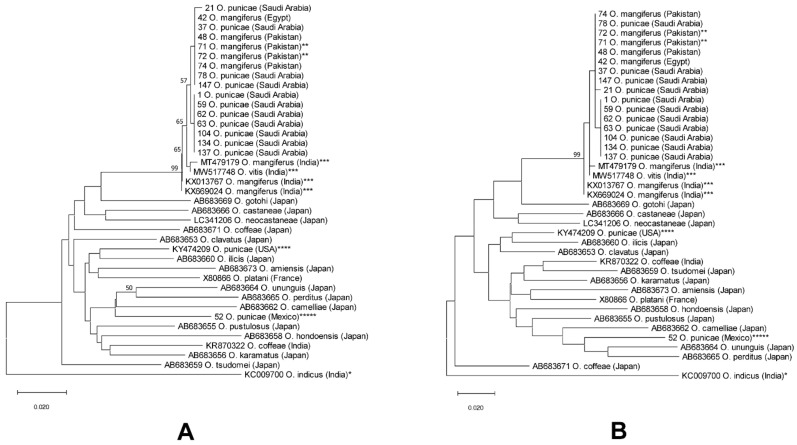
NJ (**A**) and ML (**B**) phylogenetic trees based on COI sequences of 39 spider mite samples, representing different populations of 18 closely related *Oligonychus* species. A total of 11 sequences were obtained from different hosts and regions in Saudi Arabia, one from Egypt, one from Mexico (***** claimed as *O. punicae* in Mexico), and four from Pakistan (** including two samples of *O. mangiferus* collected from the exact locality whence the original type was previously collected and described for the first time) [9]. Whereas 21 COI sequences of various closely related *Oligonychus* species were analyzed (including *** three sequences of *O. mangiferus* and one sequence of *O. vitis* from India; **** one sequence of the claimed Californian *O. punicae*), and one sequence of *O. indicus* (* an *Oligonychus* species from the other subgenus *Reckiella* Tuttle and baker, was used as an outgroup taxon). Numbers on tree branches are bootstrap values obtained from 1000 replicates.

The COI-based NJ and ML trees showed that the clade containing the *punicae* complex members (along with *O. vitis*) received a 99% bootstrap value, indicating the monophyletic nature of the three *punicae* complex members (Figure 7). Additionally, the *punicae* complex clade of both trees divided into six haplotypes, H1 (SA), H2 (Egypt, Pakistan and SA), H3 (SA), H4 (India), H5 (India), and H6 (India) (Figure 7; Table S4).

## 4. Discussion

**Figure 8:**
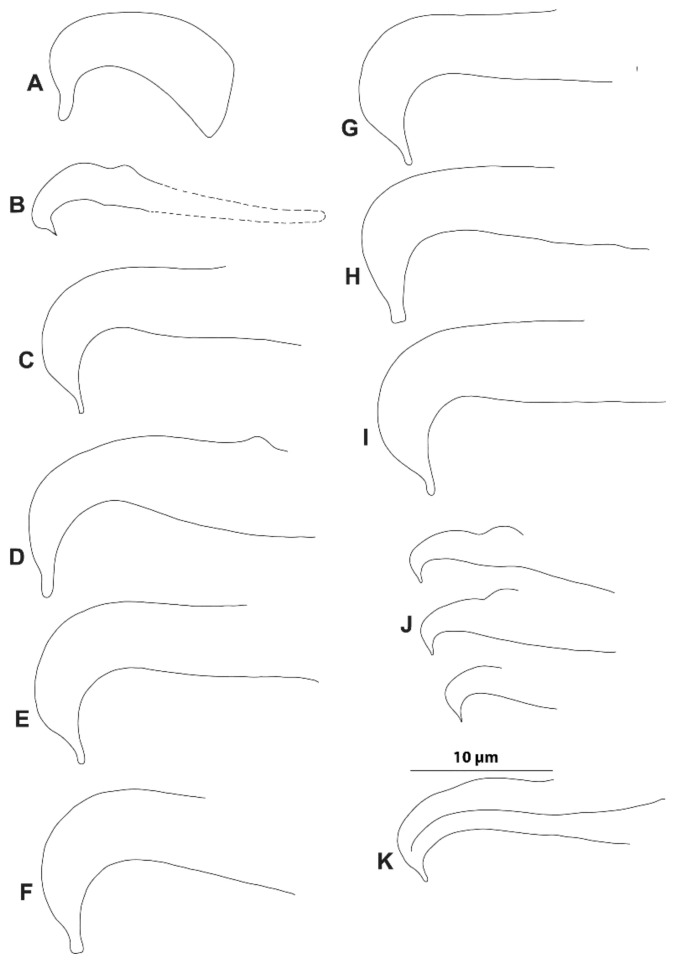
Different aedeagus shapes of *Oligonychus punicae;* (**A**), redrawn from original description [18], and redrawn from various redescriptions; (**B**) [14], (**C**) [4], (**D**) [45], (**E**) [53], (**F**) [5], (**G**) [43], (**H**) [3], (**I**) [40], (**J**) [17], and (**K**) [10].

**Figure 9:**
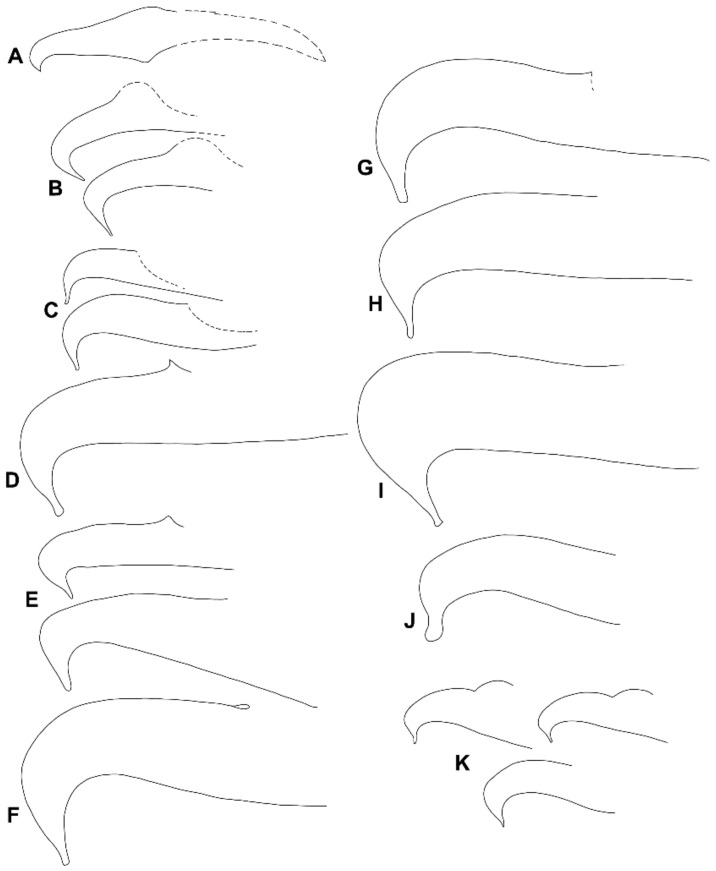
Different aedeagus shapes of *Oligonychus mangiferus;* (**A**) redrawn from original description [9], and redrawn from various redescriptions; (**B**) [14], (**C**) [4], (**D**) [5], (**E**) [7], (**F**) [5], (**G**) [3], (**H**) [25]^30^, (**I**) [42], (**J**) [41], and (**K**) [17].

**Figure 10:**
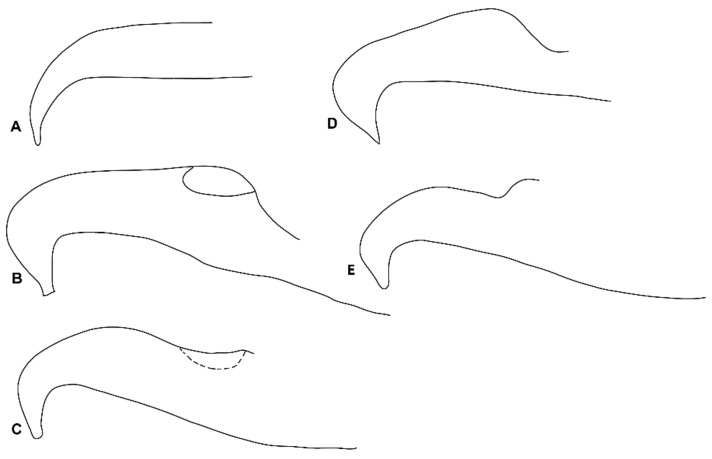
Different aedeagus shapes of *Oligonychus vitis;* (**A**) redrawn from original description [19], and redrawn from various redescriptions; (**B**) [7], (**C**) [3], (**D**) [13] and (**E**) [17].

Our results showed no reliable differentiation in the aedeagal parameters among all the various samples of the *punicae* complex collected from Egypt, Pakistan, and SA (Figure 4; Table S2). However, the relative height of the bent aedeagal part (i.e., H vs. L and W) was consistently different in the claimed Mexican *O. punicae* (Table S2; Figure 5). The three members (*O. punicae, O. mangiferus* and *O. vitis*) of the *punicae* complex have only been distinguished by slight differences in the aedeagal characters, which can be contradictory and variable among different previous descriptions and/or illustrations of *O. punicae* (Figure 8; Table S5a)*, O. mangiferus* (Figure 9; Table S5b) and *O. vitis* (Figure 10; Table S5c) from various geographical locations [3,4,5,7,9,10,12,13,14,17,18,19,25,40,42,45,51,52,53] and considered variations in the present study.

Therefore, we suggest synonymizing *O. mangiferus* with *O. punicae*, based on the observed *O. mangiferus* specimens (voucher no.: 71, 72; Figure 4E; Table S2) collected from the exact locality whence the original type was previously collected and described for the first time [9]. The identity of *O. mangiferus* has remained ambiguous since its discovery due to the limited original description/illustration, with the key character of the male aedeagus being described briefly (Figure 9A) [9]. The original aedeagal illustration (Figure 9A) of *O. mangiferus* is vague [9], especially when compared to the male aedeagus of the specimens (Figure 4E) collected from the exact locality whence the original type was previously collected and described for the first time [9]. Moreover, *O. mangiferus* was described as new to science [9], without any comparison to closely related species being provided, e.g., *O. punicae* [18]. More recently, several authors have reported *O. punicae* and *O. mangiferus* as valid species either alone or together in the same publication [3,4,5,7,10,11,12,13,14,17,25,40,42,43,45,51,52,53]. However, *O. mangiferus* has not been re-described from its type locality and the original description was overlooked. In the present study, the male aedeagus of *O. mangiferus* is illustrated (Figure 4E) using the specimens collected from the exact locality whence the original type was previously collected and described for the first time [9] and it is quite similar to the original illustration (Figure 8A) of *O. punicae* [18].

The molecular data obtained from the ITS2 and COI sequences support the synonymy of *O. mangiferus* with *O. punicae*. The obtained intraspecific range (0% to 2.1%;) of the nucleotide diversity using the ITS2 sequences of the *punicae* complex from Egypt, Israel, India, Pakistan, and SA, aligns well with previous findings on different tetranychid species [29,31,34,35,54]. Previously, the ITS2-intraspecific sequence divergence ranged between 0% and 2% (≤2%) in various tetranychid species, including the three *Oligonychus* species of *O. afrasiaticus* (McGregor)*, O. mangiferus*, and *O. perseae* (Tuttle, Baker and Abbatiello). Moreover, a 0% to 0.4% [54] and 0.2% to 2.5% [31] intraspecific ITS2-sequence divergence was detected among various populations of *Mononychellys progresivus* (Dorest) and *Eutetranychus orientalis* (Klein), respectively.

In our research, the intraspecific COI-sequence divergence among the *punicae* complex samples from Egypt, India, Pakistan, and SA is in agreement with previous tetranychid mite studies [28,30,33,54]. For example, the COI-based intraspecific sequence divergence ranged from 0% to 2.9% for 17 different *Oligonychus* species [28]. Furthermore, a 0% to 0.2% [54] and 0% to 5% [30] intraspecific COI sequence divergence was observed among various populations of *M. progresivus* and in different *Tetranychus* species, respectively.

In the present study, the COI-based molecular data revealed that *O. vitis* could be another junior synonym of *O. punicae*. The retrieved COI sequence of *O. vitis* (GenBank accession no.: MW517748, India) showed very low genetic divergence (0.3% to 0.6%) when compared with the other 19 COI sequences of *O. punicae*/*O. mangiferus* from Egypt, India, Pakistan, and SA. This limited variation falls within the range of intraspecific genetic divergence, as previously observed in various tetranychid species [28,30,33,35]. This sequence/sample of *O. vitis* was collected from the same location in India [55] as the type locality of *O. punicae* [18].

In the original description of *O. vitis* [19], the authors compared it with a re-description of *O. mangiferus* from African samples [51]. Although the male of *O. vitis* was described, the remarks were mostly based on the female holotype [19]. Additionally, it is questionable whether the original aedeagus illustration (Figure 10A) of *O. vitis* was drawn in the proper lateral position [19], causing the diagram to show greater dissimilarity from the later re-descriptions/illustrations (Figure 10B–E), either reported from Africa [3,7] or India [13,17]. Meyer [3] differentiated *O. vitis* from *O. mangiferus*/*O. punicae* based on two aedeagal parameters, namely the angle (α) formed between the bent part and shaft, and the relative height of the bent part (H) to the length (L) of the shaft dorsal margin. Our observations showed that these two aedeagal parameters are variable within the *punicae* complex, and provide insufficient detail to separate *O. mangiferus/O. punicae* from *O. vitis.* This conclusion is supported by the various samples of the *punicae* complex (Figure 4; Table S2) when compared with the previously described aedeagus for *O. punicae* (Figure 10; Table S5a), *O. mangiferus* (Figure 9; Table S5b), and *O. vitis* (Figure 10; Table S5c).

The morphological data also exposed a cryptic *Oligonychus* species that is claimed as *O. punicae* in Mexico [37]. Based on the aedeagus morphology, this Mexican cryptic *Oligonychus* sp. is different (Figure 5) from all the other examined samples of the *punicae* complex from Egypt, Pakistan, and SA (Figure 4; Table S2). This finding is supported by all previous illustrations of *O. punicae, O. mangiferus*, and *O. vitis* (Figure 8, Figure 9 and Figure 10; Table S5) [3,4,7,9,10,12,13,14,17,18,19,25,40,42,45,51,52,53]. However, based uniquely on the relative length of the bent aedeagal part to the shaft dorsal margin, the aedeagus shape (Figure 8F) illustrated for the *O. punicae* population from California [5] shows a reasonable similarity to the aedeagus of the Mexican cryptic *Oligonychus* sp. in our study (Figure 5).

In addition, the molecular data of ITS2-rDNA and COI-mtDNA confirmed that the Mexican cryptic *Oligonychus* sp. does not belong to the *punicae* complex (Figure 6 and Figure 7; Tables S3 and S4). The obtained interspecific range of genetic divergence of ITS2 and COI (17.2% to 18.8%, and 9.2% to 10.2%, respectively) that was detected between *O. punicae/O. mangiferus/O. vitis* (from Egypt, Israel, India, Pakistan, and SA) and the Mexican cryptic *Oligonychus* sp., is in accordance with separate species in previous molecular studies on tetranychid mites [28,29]. The ITS2-based interspecific nucleotide divergence ranged from 4.4% to 54.8% and has been observed previously in various tetranychid species including the three *Oligonychus* species of *O. afrasiaticus*, *O. perseae*, and *O. mangiferus* [29], whereas the COI-based interspecific sequence divergence among the 17 different *Oligonychus* species ranged from 7.3% to 18.3% [28]. In the present study, the interspecific nucleotide divergence ranged from 12.2% to 13.8% (ITS2, Table S3) and 6.1% to 11.8% (COI, Table S4) between/among different closely related *Oligonychus* species, excluding the members of the *punicae* complex.

Additionally, based on our molecular analyses of DNA sequences of ITS2 and COI available in GenBank, *O. punicae*, *O. coffeae*, and *O. ununguis* sequences seem doubtful. For example, the COI sequence (Accession no.: KY474209; Figure 7) of the claimed *O. punicae* from the USA is highly divergent (8.3%, Table S4) from the claimed Mexican *O. punicae* (voucher no. 52, Table S1; Figure 7), which we propose as a cryptic *Oligonychus* sp. Similarly, the *ununguis* complex (ITS2, Table S3; Figure 6) and the *coffeae* complex (COI, Table S4; Figure 7) need further work to be resolved. The ITS2 sequence (Accession no.: HQ709242) of *O. ununguis* from China is highly divergent (11.5%; Table S3) from the ITS2 sequence (Accession no.: JF774179) of *O. ununguis* from Korea. In addition, the COI sequence (Accession no.: AB683671) of *O. coffeae* from Japan is highly divergent (8.9%; Table S4) from the COI sequence (Accession no.: KR870322) of *O. coffeae* from India.

## 5. Conclusions

In conclusion, the morphological and molecular data of the present study resolved the taxonomic problem of the *punicae* species complex by suggesting *O. mangiferus* and *O. vitis* as junior synonyms of *O. punicae*. Moreover, the obtained morphological and molecular data propose the claimed Mexican *O. punicae* [37] as a cryptic *Oligonychus* sp. that needs to be re-identified. The Mexican cryptic *Oligonychus* sp. should undergo a taxonomic revision as it is morphologically and genetically distinct from members of the *punicae* complex (*O. punicae, O. mangiferus*, and *O. vitis*). We also propose a revision of the Californian populations of the claimed *O. punicae* to confirm their taxonomic identity. Furthermore, we emphasize that the aedeagal parameters of the height of the bent part (H), the length of the shaft dorsal margin (L), and shaft width (W) are important aedeagal characteristics for delineating the closely related *Oligonychus* species. Finally, the present study highlighted the importance of the integrated taxonomic approaches in solving the problems related to species complexes. More studies should be conducted to resolve other species complexes of the genus *Oligonychus*, e.g., *O. coffeae* and *O. ununguis.*

## Data Availability

All necessary data is provided in this paper.

## Supplementary Materials

The following supporting information can be downloaded at: https://www.mdpi.com/article/10.3390/insects14010003/s1, Table S1: Geographical distribution, host plant, collection details, ITS2/COI fragment size and GenBank accession numbers of 40 spider mite samples of the *punicae* species complex collected from Egypt, Mexico, Pakistan & Saudi Arabia and analyzed morphologically/molecularly in the present study; Table S2: Relatively measured morphometric data obtained from different aedeagal parameters—viz. height of bent aedeagal part (H), length of shaft dorsal margin (L), shaft width (W), and the angle formed between shaft axis and axis of the bent part (α) of different spider mite samples of the *punicae* species complex collected from Egypt, Mexico, Pakistan and Saudi Arabia, in the present study; Table S3: Genetic divergence (pairwise p-distance) based on ITS2 sequences, either obtained in the present study or retrieved from GenBank, among various spider mite samples, representing different populations of four closely related *Oligonychus* species, reported from different countries; Table S4: Genetic divergence (pairwise p-distance) based on COI sequences, either obtained in the present study or retrieved from GenBank, among various spider mite samples, representing different populations of 18 closely related *Oligonychus* species, reported from different countries; Table S5: Relatively measured morphometric data obtained from different aedeagal parameters—viz. height of bent aedeagal part (H), length of shaft dorsal margin (L), shaft width (W), and the angle formed between shaft axis and axis of the bent part (α) of different populations of *Oligonychus punicae*, *O. mangiferus* and *O. vitis*, previously described from various geographical localities/countries.
