# Supplementary material for: Evidence for Reconsidering the Taxonomic Status of Closely Related Oligonychus Species in punicae Complex (Acari: Prostigmata: Tetranychidae)
Source: Insects. 2022 Dec 21;14(1):3. doi: 10.3390/insects14010003 (PMC9864387; doi:10.3390/insects14010003)
Supplement: Supplementary file 1 [file insects-14-00003-s001.zip › insects-2065171-supplementary.pdf]

Evidence for Reconsidering the Taxonomic Status of Closely Related *Oligonychus* Species in *punicae* Complex (Acari: Prostigmata: Tetranychidae)

Hafiz Muhammad Saqib Mushtaq [1], Muhammad Kamran [1], Amgad A. Saleh [2], Fahad Jaber Alatawi [1,*]

[1] Acarology Research Laboratory, Department of Plant Protection, College of Food and Agriculture Sciences, King Saud University, P.O. Box No. 2460, Riyadh 11451, Saudi Arabia; hmushtaq@ksu.edu.sa (H.M.S.M.); murafique@ksu.edu.sa (M.K.)

[2] Plant Pathology Laboratory, Department of Plant Protection, College of Food and Agriculture Sciences, King Saud University, P.O. Box No. 2460, Riyadh 11451, Saudi Arabia; amgsaleh@ksu.edu.sa

* Correspondence: falatawi@ksu.edu.sa

Number of supplementary tables: 5

Table S1. Geographical distribution, host plant, collection details, ITS2/COI fragment size and GenBank accession numbers of 40 spider mite samples of the *punicae* species complex collected from Egypt, Mexico, Pakistan & Saudi Arabia and analyzed morphologically/molecularly in the present study.

**Sample voucher no.**	**Collection date**	**Locality, Province, Country**	**Host Plant**	**Collector name (Reference)**	**GPS**	**GenBank accession numbers**		**ITS2 fragment size (bp)**	**COI fragment size (bp)**
						**ITS2**	**COI**		
1	09 May 2018	Wadi Dawasir, Riyadh, Saudi Arabia	*Mangifera indica* L. (Anacardiaceae)	HMS Mushtaq, JS Basahih & M Kamran	20°27'3.56''N, 44°50'6.95''E	MZ435892	MZ425481	497	408
5	10 May 2018	Abu Arish, Jizan, Saudi Arabia	*Conocorpus erectus* L. (Combretaceae)	HMS Mushtaq, JS Basahih & M Kamran	16°59'4.09''N, 42°49'6.75''E	MZ435893	N/A	497	N/A
6	07 May 2017	Al-Ula, Madinah, Saudi Arabia	*M. indica*	EM Khan	26°40'5.86''N, 37°53'4.77''E	MZ435894	N/A	497	N/A
13	09 May 2018	Wadi Dawasir, Riyadh, Saudi Arabia	Unknown	HMS Mushtaq, JS Basahih & M Kamran	20°25'1.99''N, 44°43'5.55''E	MZ435895	N/A	497	N/A
14	20 May 2018	Al-Ula, Madinah, Saudi Arabia	*M. indica*	HMS Mushtaq, JH Mirza & M Kamran	26°51'0.94''N, 37°57'9.47''E	MZ435896	N/A	494	N/A
21	08 May 2018	Hariq, Riyadh, Saudi Arabia	*Punica granatum* L. (Lythraceae)	HMS Mushtaq, JS Basahih & M Kamran	23°37'8.43''N, 46°31'9.42''E	MZ435897	MZ425482	497	408
26	02 May 2018	Unayzah, Qassim, Saudi Arabia	*M. indica*	HMS Mushtaq, JH Mirza & M Kamran	25°51'02.6''N, 44°13'09.4''E	MZ435898	N/A	456-460	N/A
27	09 May 2018	Wadi Dawasir, Riyadh, Saudi Arabia	*P. granatum*	HMS Mushtaq, JS Basahih & M Kamran	20°27'8.29''N, 44°52'1.37''E	MZ435899	N/A	497	N/A
37	09 Mar 2019	Diriyah, Riyadh, Saudi Arabia	*C. erectus*	HMS Mushtaq	24°45'35.0''N, 46°33'56.7''E	MZ435900	MZ425483	497	410
42	06 May 2019	Al Badari, Asyut, Egypt	*M. indica*	FA Marei	26°59'18.7''N, 31°23'36.8''E	MZ435901	MZ425484	497	410
43	12 Apr 2019	Diriyah, Riyadh, Saudi Arabia	*C. erectus*	HMS Mushtaq	24°44'51.6''N, 46°31'43.5''E	MZ435902	N/A	489	N/A
44	26 May 2018	Dumah Al-Jandal, Jouf, Saudi Arabia	*Vitis vinifera* L. (Vitaceae)	HMS Mushtaq, JH Mirza & M Kamran	29°47'4.32''N, 39°52'6.80''E	MZ435903	N/A	473	N/A
48	16 Aug 2019	Daira Din Panah, Punjab, Pakistan	*M. indica*	M Iftikhar	30°34'31.7''N, 70°57'14.3''E	MZ435904	MZ425485	497	410
52*	09 Oct 2019	Chiautla, Estado de México, Mexico	*Alnus jorulensis* Kunth (Betulaceae)	MT Santillán-Galicia	19°32'33.1''N, 98°52'33.1''W	MZ435920	OP825124	429	403-410
56	01 Nov 2019	Qunfudhah, Makkah, Saudi Arabia	*C. erectus*	HMS Mushtaq, & M Kamran	19°07'58.5''N, 41°04'36.2''E	MZ435905	N/A	470	N/A
59	01 Nov 2019	Qunfudhah, Makkah, Saudi Arabia	*M. indica*	HMS Mushtaq, & M Kamran	19°23'4.45''N, 41°03'7.82''E	MZ435906	MZ425486	497	408
62	13 Apr 2019	Uyaynah, Riyadh, Saudi Arabia	*C. erectus*	HMS Mushtaq	24°53'13.9''N, 46°19'26.5''E	MZ435907	MZ425487	425	410
63	13 Apr 2019	Jubaylah, Riyadh, Saudi Arabia	*C. erectus*	HMS Mushtaq	24°53'49.2''N, 46°26'54.3''E	MZ435908	MZ425488	428	410
64	12 Apr 2019	Wadi Hanifah, Riyadh, Saudi Arabia	*C. erectus*	HMS Mushtaq	24°43'4.20''N, 46°34'3.68''E	MZ435909	N/A	463	N/A
70	22 May 2018	Tabuk, Tabuk, Saudi Arabia	*P. granatum*	HMS Mushtaq, JH Mirza & M Kamran	28°27'24.8''N, 36°33'34.9''E	MZ435910	N/A	497	N/A
71**	12 Nov 2019	Faisalabad, Punjab, Pakistan	*M. indica*	MH Bashir, EM Khan	31°25'44.0''N, 73°04'19.4''E	MZ435911	MZ425489	430	410
72**	12 Nov 2019	Faisalabad, Punjab, Pakistan	*V. vinifera*	MH Bashir, EM Khan	31°25'44.0''N, 73°04'19.4''E	MZ435912	MZ425490	444	410
73	08 Nov 2019	Sargodha, Punjab, Pakistan	*P. granatum*	M. Afzal	32°08'2.7''N, 72°41'36.2''E	MZ435913	N/A	465	N/A
74	08 Nov 2019	Sargodha, Punjab, Pakistan	*M. indica*	M Afzal	32°08'2.7''N, 72°41'36.2''E	MZ435914	MZ425491	445	410
75	08 Nov 2019	Sargodha, Punjab, Pakistan	*V. vinifera*	M Afzal	32°08'2.7''N, 72°41'36.2''E	N/A	N/A	N/A	N/A
76	20 May 2018	Al-Ula, Madinah, Saudi Arabia	*P. granatum*	HMS Mushtaq, JH Mirza & M Kamran	26°05'2.24''N, 37°58'2.22''E	MZ435915	N/A	493	N/A
77	20 May 2018	Al-Ula, Madinah, Saudi Arabia	*P. granatum*	HMS Mushtaq, JH Mirza & M Kamran	26°51'0.94''N, 37°57'9.47''E	MZ435916	N/A	468	N/A
78	22 May 2018	Tabuk, Tabuk, Saudi Arabia	*P. granatum*	HMS Mushtaq, JH Mirza & M Kamran	28°26'13.2''N, 36°39'06.7''E	MZ435917	MZ425492	446	410

80	29 Oct 2019	Bashayer, Asir, Saudi Arabia	*V. vinifera*	HMS Mushtaq, & M Kamran	19°43'4.39''N, 41°55'5.13''E	MZ435918	N/A	489	N/A
104	14 Apr 2018	Taif, Makkah, Saudi Arabia	*P. granatum*	HMS Mushtaq	21°17'3.51''N, 40°23'0.16''E	MZ435919	MZ425493	497	410
105	13 Feb 2012	Madinah, Madinah, Saudi Arabia	*C. erectus*	M Kamran [36]	24°28'7.66''N, 39°37'4.19''E	N/A	N/A	N/A	N/A
134	01 Oct 2020	Tayma, Tabuk, Saudi Arabia	*Rosa* sp. (Rosaceae)	HMS Mushtaq, JH Mirza & EM Khan	27°37'33.6"N, 38°30'48.1"E	OP821242	OP825121	497	410
137	01 Oct 2020	Tayma, Tabuk, Saudi Arabia	*P. granatum*	HMS Mushtaq, JH Mirza & EM Khan	27°38'00.5"N, 38°33'51.0"E	OP821243	OP825122	497	410
147	08 Oct 2020	Aridhah, Jizan, Saudi Arabia	*M. indica*	HMS Mushtaq, JH Mirza & EM Khan	17°02'25.9"N, 43°02'42.8"E	OP821244	OP825123	496	410
178	26 Oct 2018	Al Badari, Asyut, Egypt	*M. indica*	SA Abdelgayed	26°58'45.9"N, 31°26'18.4"E	N/A	N/A	N/A	N/A
180	24 Jul 2018	Sahel Selim, Asyut, Egypt	*M. indica*	SA Abdelgayed	27°04'03.6"N, 31°20'32.9"E	N/A	N/A	N/A	N/A
181	15 Aug 2018	Sahel Selim, Asyut, Egypt	*V. vinifera*	SA Abdelgayed	27°04'03.6"N, 31°20'32.9"E	N/A	N/A	N/A	N/A
183	05 May 2018	Badari, Asyut, Egypt	*M. indica*	SA Abdelgayed	26°58'45.9"N, 31°26'18.4"E	N/A	N/A	N/A	N/A
184	23 Apr 2022	DG Khan, Punjab, Pakistan	*M. indica*	M Kamran	29°50'39.2"N, 70°29'15.8"E	OP821245	N/A	430	N/A
185	26 Apr 2022	DG Khan, Punjab, Pakistan	*M. indica*	M Kamran	29°50'39.2"N, 70°29'15.8"E	OP821246	N/A	430	N/A

* It represents the Mexican *Oligonychus* sp. that was claimed as *O. punicae* in Mexico [37], which does not belong to the *punicae* species complex, as revealed in the present study.

** It represents the samples of *O. mangiferus* collected from the exact locality whence the original type was previously collected and described for the first time in Pakistan [9] and analyzed in the present study.

Table S2. Relatively measured morphometric data obtained from different aedeagal parameters — viz. height of bent aedeagal part (H), length of shaft dorsal margin (L), shaft width (W), and the angle formed between shaft axis and axis of the bent part (α) of different spider mite samples of the *punicae* species complex collected from Egypt, Mexico, Pakistan and Saudi Arabia, in the present study.

Sample voucher no.	No. of specimens observed (n)	H/L[1]	H/W[2]	α^3
1	1	0.50	1.30	79°
5	1	0.60	1.60	62°
6	N/A	N/A	N/A	N/A
13	1	0.50	1.10	70°
14	2	0.34–0.49	0.71–1.11	65°–78°
21	1	0.26	0.65	74°
26		0.46	1.20	68°
27	N/A	N/A	N/A	N/A
37	1	0.50	1.20	63°
42	3	0.50	0.90–1.40	63°–70°
43	2	0.50	1.30–1.50	69°–70°
44	1	0.38	0.90	74°
48	1	0.40	1.00	77°
52*	5	0.93–1.30	1.80–2.50	57° – 87°
56	1	0.37	0.92	74°
59	1	0.43	0.81	68°
62	1	0.50	1.20	70°
63	1	0.60	1.60	69°
64	1	0.47	0.94	76°
70	N/A	N/A	N/A	N/A
71**	2	0.40	1.30	65°–76°
72**	N/A	N/A	N/A	N/A
73	1	0.50	1.10	65°
74	N/A	N/A	N/A	N/A
75	N/A	N/A	N/A	N/A
76	1	0.31	0.90	73°
77	1	0.63	0.81	69°
78	N/A	N/A	N/A	N/A
80	2	0.32–0.34	0.80–1.30	70°
104	N/A	N/A	N/A	N/A
105	1	0.50	1.40	64°
134	N/A	N/A	N/A	N/A
137	N/A	N/A	N/A	N/A
147	N/A	N/A	N/A	N/A
178	1	0.46	1.00	63°
180	1	0.51	1.3	61°
181	1	0.29	0.64	66°
183	3	0.42–0.60	0.97–1.64	58°–73°

184	N/A	N/A	N/A	N/A
185	N/A	N/A	N/A	N/A

[1]**H/L,** The height of bent aedeagal part (H) divided by the length of shaft dorsal margin (L).
[2]**H/W,** The height of bent aedeagal part (H) divided by the shaft width (W).
[3]**α**, The angle formed between shaft axis and axis of the bent aedeagal part.
*It represents the Mexican *Oligonychus* sp., previously claimed as *O. punicae* in Mexico [37], which does not belong to the *punicae* species complex, as revealed in the present study.
**It represents the samples of *O. mangiferus* that were collected from the exact locality whence the original type was previously collected and described for the first time in Pakistan [9] and analyzed in the present study.

Table S3. Genetic divergence (pairwise p-distance) based on ITS2 sequences, either obtained in the present study or retrieved from GenBank, among various spider mite samples, representing different populations of four closely related *Oligonychus* species, reported from different countries.

No.	Spider mite samples/species	1 (H_1)	2 (H_2)	3 (H_3)	4 (H_4)	5	6	7	8
1	*O. punicae*/*O. mangiferus* (**H_1**)*	0.000							
2	*O. mangiferus* (**H_2**) (EF433286, Israel)	0.003	0.000						
3	*O. mangiferus* (**H_3**) (MN969994, India)	0.010	0.008	0.000					
4	*O. mangiferus* (**H_4**) (KC283029, India)	0.016	0.013	0.021	0.000				
5	*O. ununguis* (HQ709242, China)	0.125	0.128	0.130	0.138	0.000			
6	*O. ununguis* (JF774179, Korea)	0.120	0.122	0.130	0.135	0.115	0.000		
7	*O. coffeae* (AY750706, Taiwan)	0.143	0.143	0.151	0.156	0.138	0.122	0.000	
8	*O. punicae*** (Mexico)	0.172	0.174	0.182	0.188	0.151	0.125	0.164	0.000

*H1 represents the ITS2 haplotype 1 that contains 34 samples collected from Egypt, Israel, Pakistan, and Saudi Arabia (voucher no: 1, 5, 6, 13, 14, 21, 26, 27, 37, 42, 43, 44, 48, 56, 59, 62, 63, 64, 70, 71, 72, 73, 74, 76, 77, 78, 80, 104, 134, 137, 147, 184, 185; Table S1 & accession no: DQ656486) of the *punicae* species complex.

**The two samples of the claimed *O. punicae* from Mexico [37] (voucher no: 52; Table S1 & accession no: KC352302) that needs to be re-identified.

- The yellow highlighted section represents four different haplotypes of the *punicae* species complex.

Table S4. Genetic divergence (pairwise p-distance) based on COI sequences, either obtained in the present study or retrieved from GenBank, among various spider mite samples, representing different populations of 18 closely related *Oligonychus* species, reported from different countries.

No.	Spider mite samples/species	1	2	3	4	5	6	7	8	9	10	11	12	13	14	15	16	17	18	19	20	21	22	23	24
1	*O. punicae* **(H_1)***	0.000																							
2	*O. punicae* **(H_2)** (21; Saudi Arabia)	0.006	0.000																						
3	*O. punicae* / *O. mangiferus* **(H_3)****	0.003	0.003	0.000																					
4	*O. mangiferus* **(H_4)*****	0.003	0.010	0.006	0.000																				
5	*O. mangiferus* **(H_5)** (MT479179; India)	0.010	0.010	0.006	0.006	0.000																			
6	*O. vitis* **(H_6)** (MW517748; India)	0.006	0.006	0.003	0.003	0.003	0.000																		
7	*O. punicae* (KY474209, USA)****	0.086	0.092	0.089	0.083	0.089	0.086	0.000																	
8	*O. punicae* (52, Mexico)*****	0.096	0.102	0.099	0.092	0.099	0.096	0.083	0.000																
9	*O. ununguis* (AB683664, Japan)	0.102	0.108	0.105	0.099	0.105	0.102	0.089	0.080	0.000															
10	*O. gotohi* (AB683669, Japan)	0.067	0.073	0.070	0.064	0.070	0.067	0.076	0.073	0.080	0.000														
11	*O. coffeae* (AB683671, Japan)	0.080	0.086	0.083	0.076	0.080	0.080	0.064	0.102	0.102	0.089	0.000													
12	*O. coffeae* (KR870322, India)	0.099	0.105	0.102	0.096	0.102	0.099	0.083	0.080	0.092	0.083	0.089	0.000												
13	*O. castaneae* (AB683666, Japan)	0.080	0.086	0.083	0.076	0.083	0.080	0.080	0.086	0.061	0.070	0.089	0.076	0.000											
14	*O. pustulosus* (AB683655, Japan)	0.086	0.092	0.089	0.083	0.089	0.086	0.061	0.083	0.083	0.076	0.070	0.061	0.080	0.000										
15	*O. tsudomei* (AB683659, Japan)	0.086	0.092	0.089	0.083	0.089	0.086	0.073	0.083	0.102	0.083	0.092	0.067	0.089	0.076	0.000									
16	*O. ilicis* (AB683660, Japan)	0.086	0.092	0.089	0.083	0.083	0.086	0.054	0.083	0.083	0.070	0.070	0.096	0.086	0.076	0.092	0.000								
17	*O. amiensis* (AB683673, Japan)	0.102	0.108	0.105	0.099	0.105	0.102	0.089	0.105	0.089	0.089	0.096	0.096	0.080	0.083	0.092	0.076	0.000							
18	*O. hondoensis* (AB683658, Japan)	0.111	0.118	0.115	0.108	0.115	0.111	0.086	0.105	0.099	0.115	0.096	0.086	0.086	0.080	0.099	0.099	0.105	0.000						
19	*O. perditus* (AB683665, Japan)	0.111	0.118	0.115	0.108	0.115	0.111	0.096	0.080	0.073	0.067	0.105	0.096	0.086	0.089	0.092	0.092	0.108	0.118	0.000					
20	*O. clavatus* (AB683653, Japan)	0.076	0.083	0.080	0.073	0.080	0.076	0.054	0.092	0.099	0.067	0.080	0.080	0.070	0.070	0.076	0.064	0.089	0.086	0.096	0.000				
21	*O. neocastaneae* (LC341206, Japan)	0.076	0.083	0.080	0.073	0.080	0.076	0.083	0.096	0.102	0.073	0.086	0.086	0.064	0.083	0.086	0.083	0.108	0.111	0.108	0.067	0.000			
22	*O. karamatus* (AB683656, Japan)	0.092	0.099	0.096	0.089	0.096	0.092	0.064	0.076	0.096	0.076	0.070	0.067	0.073	0.070	0.083	0.067	0.086	0.089	0.086	0.067	0.070	0.000		
23	*O. camelliae* (AB683662, Japan)	0.115	0.115	0.111	0.111	0.105	0.108	0.083	0.083	0.083	0.086	0.092	0.092	0.080	0.083	0.089	0.076	0.092	0.096	0.086	0.086	0.102	0.089	0.000	

**24**	*O. platani* (X80866, France)	0.096	0.102	0.099	0.092	0.099	0.096	0.073	0.099	0.099	0.092	0.092	0.083	0.080	0.073	0.083	0.064	0.086	0.092	0.102	0.070	0.083	0.073	0.080	0.000

* H1 represents the COI haplotype 1 that contains seven samples of *O. punicae* collected from Saudi Arabia (voucher no: 1, 59, 62, 63, 104, 134 & 137; Table S1).
** H3 represents the COI haplotype 3 that contains eight samples of the *punicae* species complex collected from Egypt, Pakistan, and Saudi Arabia (voucher no: 37, 42, 48, 71, 72, 74, 78 & 147; Table S1).
*** H4 represents the COI haplotype 4 that contains two accession numbers KX013767 and KX669024, identified as *O. mangiferus* from India.
**** The COI haplotype representing the claimed *O. punicae* from USA that needs to be re-identified.
***** The COI haplotype representing the claimed *O. punicae* from Mexico [37] (voucher no: 52; Table S1) that needs to be re-identified.
- The yellow highlighted section represents six different haplotypes of the *punicae* species complex.

Table S5. Relatively measured morphometric data obtained from different aedeagal parameters — viz. height of bent aedeagal part (H), length of shaft dorsal margin (L), shaft width (W), and the angle formed between shaft axis and axis of the bent part (α) of different populations of *Oligonychus punicae*, *O. mangiferus* and *O. vitis*, previously described from various geographical localities/countries.

**Species**	**References**	**H/L**[1]	**H/W**[2]	**α**[3]
**a)** ***O. punicae***	[18]*	0.66	1.27	52°
	[14]	0.65	1.00	55°
	[4]	0.46	1.13	85°
	[45]	0.39	0.90	80°
	[53]	0.41	1.27	78°
	[5]	0.85	1.44	80°
	[43]	0.42	1.23	92°
	[3]	0.53	1.20	98°
	[40]	0.44	1.10	92°
	[17]	0.36	0.71	81°
	[10]	0.38	0.96	86°
**b)** ***O. mangiferus***	[9]**	0.14	0.32	85°
	[14]	0.39–0.53	1.00–1.80	73°
	[4]	0.29–0.36	0.80	73°–89°
	[51]	0.43	0.81	83°
	[7]	0.29–0.31	0.75–0.64	60°
	[5]	0.39	0.86	72°
	[3]	0.38	1.10	73°
	[25]	0.34	0.80	87°
	[42]	0.30	0.83	63°
	[41]	0.26	0.75	80°
	[17]	0.33	0.71	80°
**c)** ***O. vitis***	[19]***	0.41	1.10	120°
	[7]	0.43	0.86	69°
	[3]	0.39	0.77	74°
	[13]	0.38	0.80	70°
	[17]	0.44	0.94	66°

[1]**H/L,** The height of bent aedeagal part (H) divided by length of shaft dorsal margin (L).
[2]**H/W,** The height of bent aedeagal part (H) divided by shaft width (W).
[3]**α,** The angle formed between shaft axis and axis of the bent aedeagal part.
* It represents the population of *O. punicae*, reported in the original description from India.
** It represents the population of *O. mangiferus*, reported in the original description from Pakistan.
*** It represents the population of *O. vitis*, reported in the original description from Egypt.
